# 

**Published:** 1862-12

**Authors:** 


### CONTENTS.

ORIGINAL ESSAYS AND COMMUNICATIONS.

Metals and Alloys. By Dr. B. Wood........ 533

Advancement of the Dental Profession. By Dr. A. C. Hawes........ 537

CORRESPONDENCE

New York Correspondence........ 543

SELECTIONS.

Dentition and its Derangements........ 552
Cases in Country Practice........ 558
Is Shaving favorable to Health ?—A Plea for Beards........ 560
Ether........ 568
Infants with Teeeth at Birth........ 564
To ascertain the purity of Chloroform........ 564
On the use of Log-wood in Cancer........ 564

EDITORIAL.

Theoro-Practical Hints........ 564
The American Dental Convention........ 567
Dental Association........ 568
Close of Volume........ 568
Steam Engine........ 569
Personal........ 569
Acknowledgement........ 569
To our Subscribers........ 571
Directory of Hospitals........ 571
Sherwood's New Molar Forceps........ 571
A Squib........ 570
Died........ 570

